# Brain atrophy and endovascular treatment effect in acute ischemic stroke: a secondary analysis of the MR CLEAN trial

**DOI:** 10.1177/17474930211054964

**Published:** 2021-10-28

**Authors:** Sven PR Luijten, Kars CJ Compagne, Adriaan CGM van Es, Yvo BWEM Roos, Charles BLM Majoie, Robert J van Oostenbrugge, Wim H van Zwam, Diederik WJ Dippel, Frank J Wolters, Aad van der Lugt, Daniel Bos

**Affiliations:** 1 Department of Radiology and Nuclear Medicine, Erasmus University Medical Center, Rotterdam, The Netherlands; 2 Department of Radiology, Leiden University Medical Center, Leiden, The Netherlands; 3 Department of Neurology, Amsterdam University Medical Center, Amsterdam, The Netherlands; 4 Department of Radiology and Nuclear Medicine, Amsterdam University Medical Center, Amsterdam, The Netherlands; 5 Department of Neurology, Cardiovascular Research Institute Maastricht, Maastricht University Medical Center, Maastricht, The Netherlands; 6 Department of Radiology and Nuclear Medicine, Cardiovascular Research Institute Maastricht, Maastricht University Medical Center, Maastricht, The Netherlands; 7 Department of Neurology, Erasmus Medical Center, Rotterdam, The Netherlands; 8 Department of Epidemiology, Erasmus Medical Center, Rotterdam, The Netherlands

**Keywords:** Ischemic stroke, acute stroke therapy, CT scan

## Abstract

**Background:**

Brain atrophy is suggested to impair the potential for functional recovery after acute ischemic stroke. We assessed whether the effect of endovascular treatment is modified by brain atrophy in patients with acute ischemic stroke due to large vessel occlusion.

**Methods:**

We used data from MR CLEAN, a multicenter trial including patients with acute ischemic stroke due to anterior circulation large vessel occlusion randomized to endovascular treatment plus medical care (intervention) versus medical care alone (control). We segmented total brain volume (TBV) and intracranial volume (ICV) on baseline non-contrast computed tomography (n = 410). Next, we determined the degree of atrophy as the proportion of brain volume in relation to head size (1 − TBV/ICV) × 100%, analyzed as continuous variable and in tertiles. The primary outcome was a shift towards better functional outcome on the modified Rankin Scale expressed as adjusted common odds ratio. Treatment effect modification was tested using an interaction term between brain atrophy (as continuous variable) and treatment allocation.

**Results:**

We found that brain atrophy significantly modified the effect of endovascular treatment on functional outcome (P for interaction = 0.04). Endovascular treatment led to larger shifts towards better functional outcome in the higher compared to the lower range of atrophy (adjusted common odds ratio, 1.86 [95% CI: 0.97–3.56] in the lowest tertile vs. 1.97 [95% CI: 1.03–3.74] in the middle tertile vs. 3.15 [95% CI: 1.59–6.24] in the highest tertile).

**Conclusion:**

Benefit of endovascular treatment is larger in the higher compared to the lower range of atrophy, demonstrating that advanced atrophy should not be used as an argument to withhold endovascular treatment.

## Introduction

Benefit of endovascular treatment (EVT) for acute ischemic stroke (AIS) due to large vessel occlusion (LVO) in the anterior cerebral circulation has been demonstrated among a diverse variety of patient subgroups including older adults over age 80.^1^ This group currently makes up 25% of the stroke population receiving EVT in daily clinical practice.^2^ In these patients, brain atrophy is a common finding on diagnostic stroke imaging at hospital admission.^3^ Whether brain atrophy influences the effect of EVT, however, is undetermined.

Previous studies showed that in patients receiving EVT brain atrophy is independently associated with poor functional outcome,^4,5^ but modification of the effect of EVT by brain atrophy in clinical controlled trials has not been reported. Given that the age distribution of EVT-eligible patients is shifting towards older ages^2,6^ and will continue to do so over the next years due to aging of the population,^7,8^ atrophy will be encountered more frequently.^9,10^ This may be relevant for optimizing patient selection as brain atrophy may provide more individualized estimates of expected treatment benefit and clinical outcome as opposed to age.

Therefore, we investigated whether brain atrophy modifies the effect of EVT in patients with AIS due to LVO, and particularly, whether treatment benefit is uniform across the entire range of brain atrophy.

## Methods

### Study population

Data were used from MR CLEAN,^11^ a multicenter trial including patients (n = 500) with diagnosis of AIS due to LVO in the anterior circulation presenting within 6 h after symptom onset and randomized to EVT plus medical care (intervention) versus medical care alone (control). All patients or their legal representatives provided written informed consent. Approval for the study was provided by the central ethics committee of Erasmus MC University Medical Center (MEC-2010-041). All patients were 18 years or older, the trial did not have an upper age limit for inclusion. A more detailed overview of all study procedures has been published previously.^12^ Patients with baseline non-contrast computed tomography (NCCT) scans on which brain atrophy could not be reliably assessed due to previous strokes (n = 61), image artefacts related to motion, metal, or beam hardening (n = 21), no full head coverage on CT (n = 5), structural abnormalities (meningioma/ventricular drain; n = 2), and missing CT data (n = 1) were excluded (Supplemental Figure 1).

**Figure 1.**
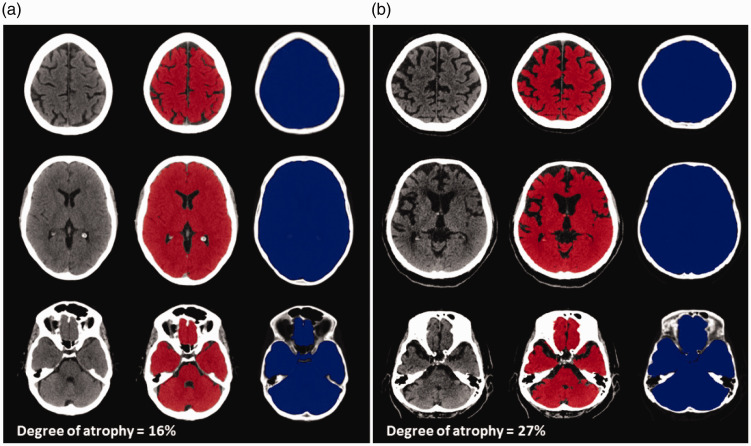
Assessment of brain atrophy. Examples of axial non-contrast CT images of two patients with total brain volume (TBV) masks shown in red and intracranial volume (ICV) masks shown in blue. (a) A 43-year-old female patient presenting with a left M2 middle cerebral artery occlusion. (b) An 81-year-old male patient presenting with a left intracranial carotid artery occlusion.

### Assessment of brain atrophy

Total brain volume (TBV) and intracranial volume (ICV) were estimated on NCCT scans using a validated automated segmentation method.^13^ In short, this method uses the segmentation algorithms implemented in SPM12 to generate probabilistic tissue maps of TBV and ICV which are subsequently binarized by applying validated optimal thresholds.^13^ In order to correct misclassification of orbital tissue as ICV, we registered an adult ICV template onto individual binary ICV masks and excluded voxels classified as ICV located outside the ICV template.^13,14^ All TBV and ICV masks were visually inspected for inaccuracies and manually corrected if needed. For each patient, TBV and ICV was calculated using the binary masks by multiplying the number of masked voxels by voxel volume and expressed in milliliters (mL). In this study, degree of brain atrophy was defined as the proportion of brain volume in relation to head size using the following equation: (1 − TBV/ICV) × 100% (Figure 1). However, normally CSF occupies part of the ICV (e.g., within sulci, basal cisterns, and ventricles) in the absence of atrophy, which inherently means that the results from the equation above will always yield a minimal value which in our study was 11%.

### Assessment of covariables

Information on baseline patient characteristics regarding medical history was obtained at admission to the hospital. Infarct extent was assessed on baseline NCCT using the Alberta Stroke Program Early Computed Tomography Score (ASPECTS).^15^ Assessment of occlusion location done on baseline vessel imaging (CTA or MRA), and collateral status on baseline CTA following a 4-point visual scale where filling of vessels in the occluded middle cerebral artery territory is compared to the unaffected hemisphere.^16^

### Primary and secondary outcomes

The primary outcome was functional outcome assessed with the modified Rankin Scale (mRS) at 90 days by an independent research nurse who was unaware of treatment allocation, and adjudicated by an independent, blinded committee.^17^ Secondary outcomes include the NIH Stroke Scale (NIHSS) score at 24 h, recanalization status on CTA at 24 h according to the Arterial Occlusive Lesion (AOL) score, and final infarct volume (FIV) on NCCT at 5–7 days using semi-automatic segmentation methods.^18,19^ In the intervention arm, recanalization status was also assessed on post-treatment digital subtraction angiography (DSA) according to the modified Treatment in Cerebral Ischemia (mTICI) score and mTICI ≥ 2B was considered as successful recanalization.^20^

### Statistical analysis

Brain atrophy (%) was analyzed as a continuous variable and, for ease of presentation, in tertiles. The association between brain atrophy and functional outcome was assessed using ordinal logistic regression. The association between brain atrophy and secondary outcomes including NIHSS at 24 h, recanalization grade, and FIV was assessed using either ordinal logistic or linear regression. FIV had a right-skewed distribution and was log-transformed to meet the assumptions for linear regression. For all regression analyses, adjustments were made for potential confounders including age, sex, smoking, diabetes mellitus, hypertension, NIHSS at baseline, time from onset to randomization, occlusion of the internal carotid artery terminus, collateral status, ASPECTS, and prestroke mRS. Treatment effect modification was evaluated by adding an interaction term between brain atrophy (as continuous variable) and treatment allocation to these models. As adding restricted cubic splines did not improve model fit (p likelihood ratio test = 0.17), a linear term for brain atrophy was used.

We conducted a sensitivity analysis where we compared brain atrophy and age as effect modifiers in order to assess whether possible heterogeneity of treatment effect by brain atrophy cannot simply be explained by age. Missing variables were imputed using multiple imputation by chained equations. Treatment effect estimates were expressed as adjusted common odds ratios (acOR) or adjusted beta values (aβ) with corresponding 95% confidence intervals (95% CI). All analyses were based on the intention-to-treat principle. Statistical analyses were performed using R statistical programming (version 3.6.1).

## Results

### Patient characteristics

In total, 410 out of 500 patients were included in the final analysis of whom 190 were assigned to the intervention arm and 220 to the control arm (Supplemental Figure 1). The median TBV was 1133 mL (interquartile range [IQR], 1054–1248), median ICV 1427 mL (IQR, 1323–1549), and median atrophy 20% (IQR, 18–22). Degree of atrophy increased with advancing age (ρ = 0.46, 95% CI: 0.38 to 0.53; Supplemental Figure 2). Per increasing tertile of brain atrophy, patients also more often reported a history of hypertension and were less often smokers (Table 1).

**Figure 2.**
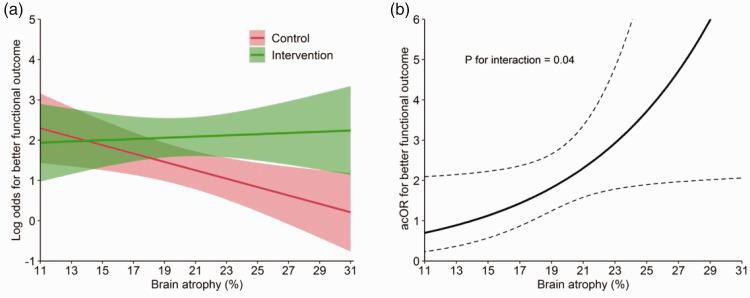
Association of brain atrophy with functional outcome and effect of endovascular treatment. Association of brain atrophy with functional outcome expressed as the log odds for better functional outcome (modified Rankin Scale [mRS], 0–6) with corresponding 95% confidence intervals (shading) stratified by treatment allocation (a). Effect of endovascular treatment with brain atrophy expressed as the adjusted common odds ratio (acOR) for a shift towards better functional outcome (mRS, 0–6) with corresponding 95% confidence interval (dashed lines) (b). Graphs were created using the fully adjusted models with all covariates fixed at their respective mean or mode.

**Table 1.** Patient characteristics per tertile of brain atrophy (%)

		Brain atrophy tertile		
	Total sample N = 410	Lowest (11–19%) N = 137	Middle (19–22%) N = 136	Highest (22–32%) N = 137
Age, median (IQR)	65 (55–76)	59 (49–66)	63 (53–73)	74 (67–81)
Male sex, no. (%)	235 (57.3)	74 (54.0)	84 (61.8)	77 (56.2)
Smoking, no. (%)	123 (30.0)	51 (37.2)	43 (31.6)	29 (21.2)
Diabetes, no. (%)	53 (12.9)	12 (8.8)	22 (16.2)	19 (13.9)
Hypertension, no. (%)	173 (42.2)	46 (33.6)	60 (44.1)	67 (48.9)
Baseline NIHSS, median (IQR)	18 (14–22)	17 (13–20)	19 (14–22)	18 (15–22)
Treated with IVT, no. (%)	371 (90.5)	126 (92.0)	119 (87.5)	126 (92.0)
Prestroke mRS ≤ 2, no. (%)	397 (96.8)	133 (97.1)	134 (98.5)	130 (94.9)
Time to randomization^a^ (min), median (IQR)	203 (150–261)	211 (157–260)	190 (140–256)	204 (163–264)
ASPECTS^b^, no. (%)				
0–5	26 (6.4)	10 (7.5)	10 (7.4)	6 (4.4)
6–10	381 (93.6)	124 (92.5)	126 (92.6)	131 (95.6)
Occlusion location, no. (%)				
ICA	4 (1.0)	0 (0.0)	2 (1.5)	2 (1.5)
ICA-T	106 (25.9)	34 (24.8)	43 (31.6)	29 (21.3)
M1	263 (64.3)	90 (65.7)	82 (60.3)	91 (66.9)
M2	33 (8.1)	10 (7.3)	9 (6.6)	14 (10.3)
A2	3 (0.7)	3 (2.2)	0 (0.0)	0 (0.0)
Collateral status^c^, no. (%)				
Absent	23 (5.7)	7 (5.2)	9 (6.7)	7 (5.1)
Poor	98 (24.3)	29 (21.6)	31 (23.1)	38 (27.9)
Moderate	169 (41.8)	64 (47.8)	51 (38.1)	54 (39.7)
Good	114 (28.2)	34 (25.4)	43 (32.1)	37 (27.2)

IQR, range; NIHSS, National Institutes of Health Stroke Scale; IVT, intravenous thrombolysis; mRS, modified Rankin Scale; ASPECTS, Alberta Stroke Program Early Computed Tomography Score.

a Time to randomization missing in one patient.

b ASPECTS missing in three patients.

c Collateral status missing in six patients.

### Primary outcome

We found that brain atrophy significantly modified the effect of EVT on functional outcome (acOR for better functional outcome per 5% atrophy increase, 0.99 [95% CI: 0.63–1.63] in the EVT plus medical care group vs. 0.60 [95% CI: 0.39–0.92] in the medical care alone group; P for interaction 0.04; Figure 2). Benefit of EVT was larger in the higher range of atrophy, with stronger shifts on the mRS in favor of EVT per increasing tertile of brain atrophy (acOR for better outcome, 1.86 [95% CI: 0.97–3.56] in the lowest tertile vs. 1.97 [95% CI: 1.03–3.74] in the middle tertile vs. 3.15 [95% CI: 1.59–6.24] in the highest tertile). In the sensitivity analysis, we found a similar significant negative association between age and functional outcome in both treatment groups (acOR for better functional outcome per 10 years increase, 0.80 [95% CI: 0.64–0.99] in the EVT plus medical care group vs. 0.60 [95% CI: 0.48–0.75] in the medical care alone group; Supplemental Figure 3B). Age did not modify the effect of EVT (P for interaction = 0.44).

**Figure 3.**
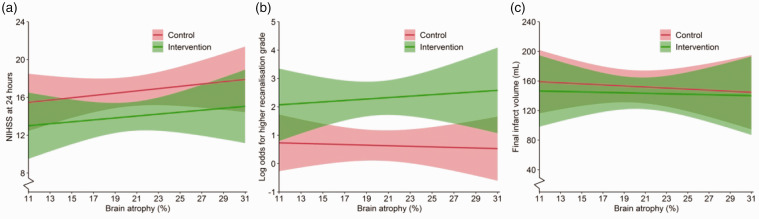
Association of brain atrophy with secondary outcomes. Association of brain atrophy with National Institutes of Health Stroke Scale (NIHSS) score at 24 h (a), recanalization grade on CTA with arterial occlusive lesion (AOL) score at 24 h (b), and final infarct volume (FIV, in milliliters [mL]) at 5–7 days (c) with corresponding 95% intervals (shading) stratified by treatment allocation. For illustration purposes, non-transformed FIV was used. Graphs were created using the fully adjusted models with all covariates fixed at their respective mean or mode.

### Secondary outcomes

Higher degree of brain atrophy correlated with higher overall NIHSS scores at 24 h, but the effect of EVT on NIHSS at 24 h did not differ significantly by degree of brain atrophy (P for interaction = 0.64; Figure 3). Recanalization rates on CTA at 24 h also favored EVT irrespective of brain atrophy (P for interaction 0.93; Figure 3). Specifically, in patients treated with EVT, successful recanalization (mTICI ≥ 2B) rates on post-procedural DSA images were similar between brain atrophy tertiles and was reached in 56.8%, 61.5%, and 63.0% of patients in the lowest, middle, and highest tertile, respectively (P = 0.95; Supplemental Table).

Median infarct volumes were 61 mL, 67 mL, and 61 mL in the lowest, middle, and highest atrophy tertile, respectively (P = 0.78; Supplemental Table). The effect of EVT on reduction of FIV was also not modified by brain atrophy (P for interaction = 0.64; Figure 3).

## Discussion

We found that brain atrophy modified the effect of EVT in patients with AIS due to LVO in the anterior circulation. Importantly, benefit of EVT was largest in the higher range of brain atrophy. Additional analyses revealed that effect of EVT on secondary outcomes including NIHSS at 24 h, recanalization on CTA, and FIV remained unchanged by brain atrophy. The heterogeneity of treatment effect on functional outcome was mainly driven by the strong decline in functional outcome with increasing atrophy in the control group as opposed to the intervention group. When degree of atrophy was low, patients were more likely to achieve good functional outcome even when EVT was not performed. These findings corroborate with the hypothesis that brain atrophy may indeed be a suitable measure of brain reserve and reflect the capacity to sustain ischemic injury.^21^ Patients with low degree of atrophy thus have high brain reserve, making them more resilient to ischemic injury which is reflected by the extent of functional recovery seen in these patients regardless of treatment that is given. It seems there may even be little benefit of EVT in terms of functional outcome in patients with <15% atrophy. However, these results should be interpreted with caution because there were few patients with very low degree of atrophy resulting in limited precision of estimates at the extreme ends of the atrophy range.

On the other hand, early neurologic recovery, quality of recanalization, and reduction of infarct volume were similar across the entire range of brain atrophy.

Previous observational studies demonstrated associations of brain atrophy with worse functional outcomes following EVT,^4,5^ which, interestingly, we could not replicate. Importantly, distinct differences in study design (i.e., retrospective observational research versus efficacy research) likely underlie these differences. In the current analysis, we used data from a randomized clinical trial thereby controlling for potential selection bias as well as confounding for which observational studies are more susceptible.

The decrease in proportional brain volume with advancing age found here is consistent with prior studies.^9,10^ A direct comparison between age and brain atrophy as effect modifiers, however, showed that heterogeneity of treatment effect is more evident for brain atrophy as opposed to age. This implies that brain atrophy is a more robust parameter for estimating expected treatment benefit in individual patients. However, instead of considering brain atrophy separately, other pre-existing imaging features such as intracranial carotid artery calcification,^22^ white matter lesions,^23^ and old ischemic infarcts^3^ are also considered indicators of brain frailty or vulnerability to ischemia. Additional work is needed to better understand the prognostic value of these imaging parameters combined and whether they can be used to aid selection of patients for EVT.^24^ Further study can also clarify whether the effects we observed on functional outcome extend to the occurrence of post-stroke cognitive impairment and dementia, in which brain reserve also plays an important role.

Strengths of this study include using data from the MR CLEAN trial including a well-defined population of patients with AIS and randomizing treatment allocation. Another strength is that we quantitatively assessed brain atrophy using validated segmentation methods. This approach may provide more precise estimations of degree of atrophy and facilitates reproducibility as opposed to visual methods to grade atrophy, which have considerable inter-observer variability.^25^ Some limitations must also be considered. Despite inclusive trial criteria without specification of an upper age limit in MR CLEAN, the proportion of elderly patients (>80 years) was lower than what is encountered in daily clinical practice (16% vs. 25%).^2,11^ Patients with advanced brain atrophy are potentially underrepresented in our sample which may affect generalizability. Also, baseline NCCT images of patients presenting with AIS were used for segmenting brain volumes. The current method does not account for expansion of brain volume or brain swelling due to early ischemic changes (e.g., edema), which in turn may lead to overestimation of TBV. To correct for this, we incorporated baseline ASPECTS and time to randomization as surrogate indicators of brain swelling due to ischemia in our regression analyses.^26^ However, since most patients had favorable baseline ASPECTS, this approach may not fully control for brain swelling.

## Conclusion

Brain atrophy within the range measured in this study modified the effect of EVT in patients with AIS due to LVO in the anterior circulation. Benefit of EVT is larger in the higher compared to the lower range of atrophy, demonstrating that advanced atrophy should not be used as an argument to withhold EVT.

## Supplemental Material

sj-pdf-1-wso-10.1177_17474930211054964 - Supplemental material for Brain atrophy and endovascular treatment effect in acute ischemic stroke: a secondary analysis of the MR CLEAN trial Click here for additional data file. Supplemental material, sj-pdf-1-wso-10.1177_17474930211054964 for Brain atrophy and endovascular treatment effect in acute ischemic stroke: a secondary analysis of the MR CLEAN trial by Sven PR Luijten, Kars CJ Compagne, Adriaan CGM van Es, Yvo BWEM Roos, Charles BLM Majoie, Robert J van Oostenbrugge, Wim H van Zwam, Diederik WJ Dippel, Frank J Wolters, Aad van der Lugt and Daniel Bos in International Journal of Stroke
