# Supplementary material for: Brain atrophy and endovascular treatment effect in acute ischemic stroke: a secondary analysis of the MR CLEAN trial
Source: Int J Stroke. 2021 Oct 28;17(8):881–8. doi: 10.1177/17474930211054964 (PMC9483187; doi:10.1177/17474930211054964)
Supplement: sj-pdf-1-wso-10.1177_17474930211054964 - Supplemental material for Brain atrophy and endovascular treatment effect in acute ischemic stroke: a secondary analysis of the MR CLEAN trial [file sj-pdf-1-wso-10.1177_17474930211054964.pdf]

Supplementary material

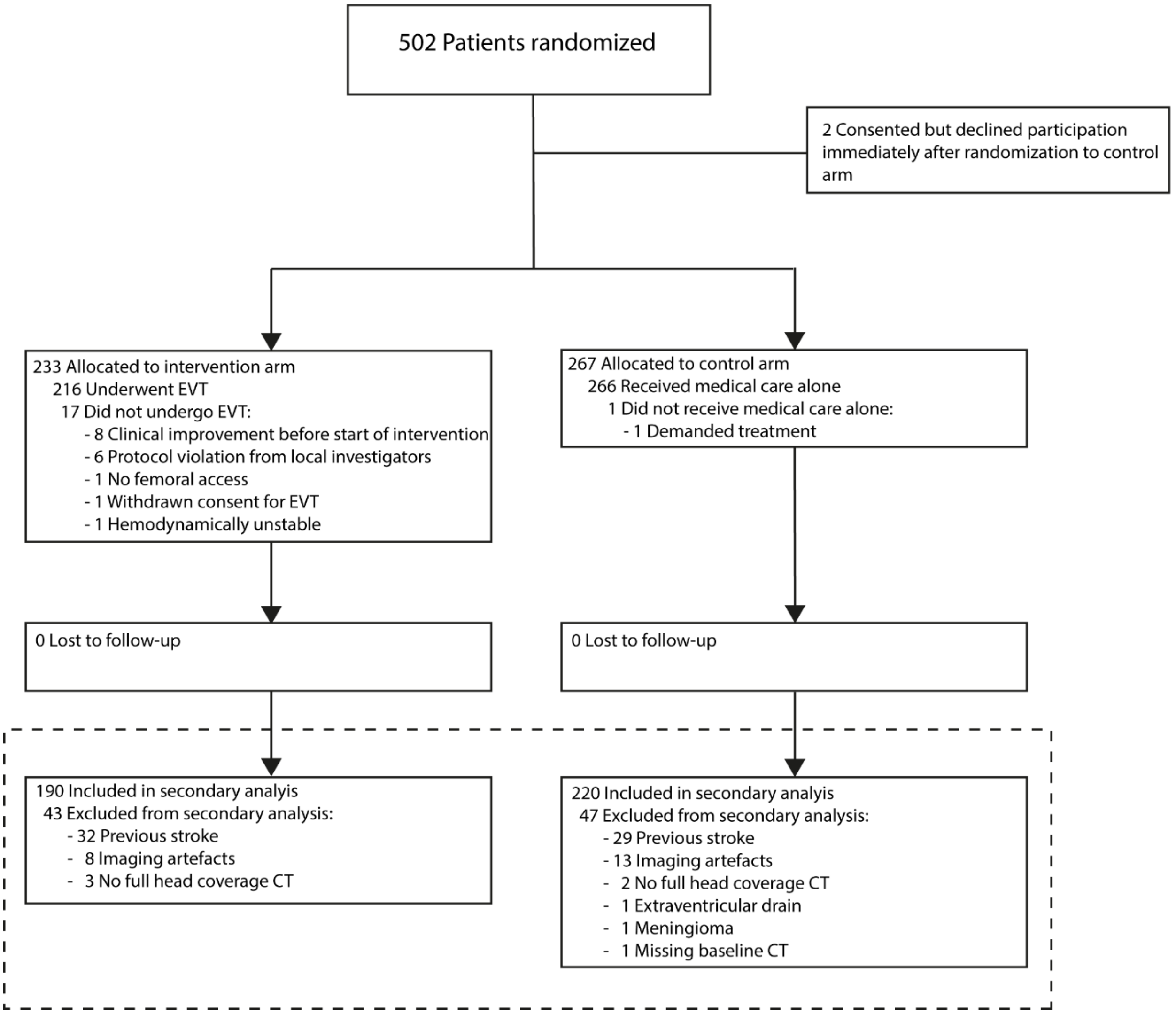

Supplementary figure 1. Patient Flow Diagram

Dashed line indicates patient inclusion for current analysis.

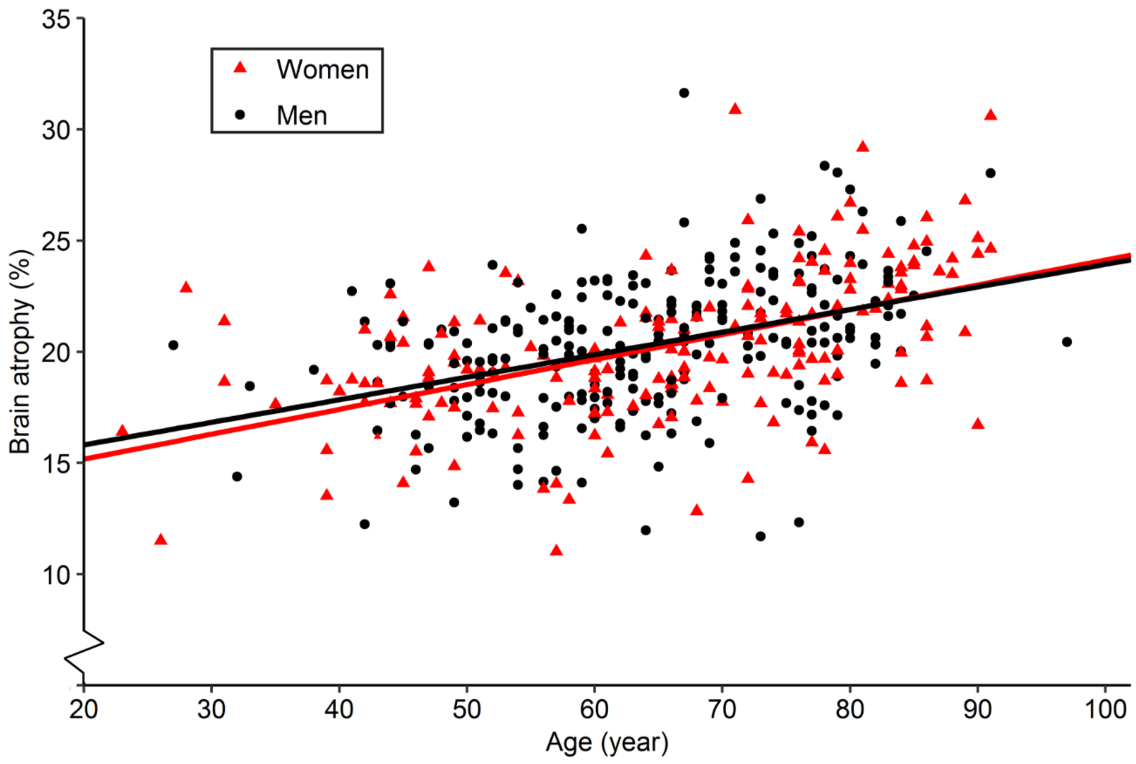

Supplementary Figure 2. Scatterplot of brain atrophy with age

Brain atrophy determined as the proportion of brain volume lost in relation to head size (1-TBV/ICV)x100% over age (in years) in total study population (n=410). Regression lines for linear fit were added according to sex.

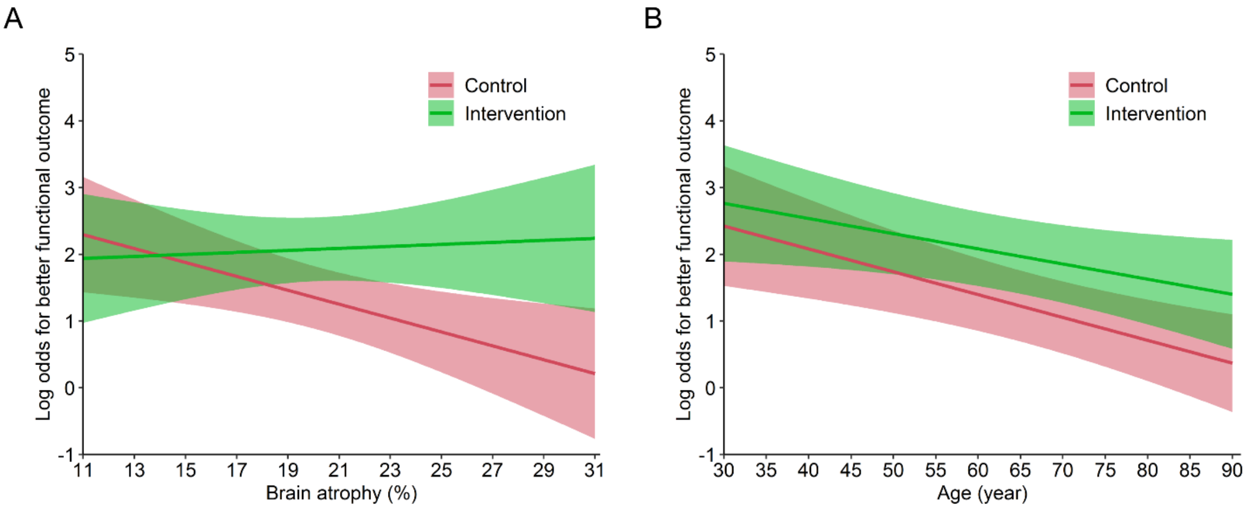

Supplementary Figure 3. Association of brain atrophy and age with functional outcome

Association of brain atrophy (A) and age (B) with functional outcome (modified Rankin Scale [mRS], 0-6) expressed as the log odds for better functional outcome with corresponding 95% confidence interval (shading) stratified by treatment allocation. Graphs were created using the fully adjusted models with all covariates fixed at their respective mean or mode.

Supplemental Table. Outcome parameters according to treatment allocation per tertile of brain atrophy (%)

	**Lowest (11-19%)**		**Middle (19-22%)**		**Highest (22-32%)**	
	Intervention N=62	Control N=75	Intervention N=63	Control N=73	Intervention N=65	Control N=72
**mRS 0-2**, No. (%)	28 (45.2)	21 (28.0)	20 (31.7)	15 (20.5)	23 (35.4)	8 (11.1)
**NIHSS at 24 hours**[a], median [IQR]	10 (4-16)	16 (10-19)	13 (6-19)	17 (13-22)	14 (5-21)	16 (14-21)
**mTICI 2B-3**[b], No. (%)	29 (56.8)	n/a	32 (61.5)	n/a	34 (63.0)	n/a
**FIV**[c], median [IQR]	50 (20-149)	75 (34-161)	42 (17-117)	78 (40-125)	56 (17-142)	75 (19-129)

mRS, modified Rankin Scale; NIHSS, National Institutes of Health Stroke Scale; mTICI, modified Thrombolysis in Ischemia; FIV, final infarct volume.

[a]NIHSS at 24 hours missing in 13 patients

[b]mTICI score missing in 33 patients treated with EVT

[c]Follow-up infarct volume missing in 31 patients
